# A non-randomized controlled stepped wedge trial to evaluate the effectiveness of a multi-level mammography intervention in improving appointment adherence in underserved women

**DOI:** 10.1186/s13012-015-0334-x

**Published:** 2015-10-14

**Authors:** L. Highfield, S.S. Rajan, M.A. Valerio, G. Walton, M.E. Fernandez, L.K. Bartholomew

**Affiliations:** Department of Management, Policy and Community Health Practice, University of Texas School of Public Health, Houston, TX USA; Department of Health Promotion and Behavioral Sciences, University of Texas School of Public Health, Houston, TX USA; Breast Health Collaborative of Texas, Houston, TX USA

**Keywords:** Breast cancer screening, Underserved women, Evidence-based interventions, Patient navigation, Mammogram adherence, Non-randomized controlled trial, Budget impact analysis

## Abstract

**Background:**

Considerable racial and socio-economic disparities exist in breast cancer. In spite of the existence of numerous evidence-based interventions (EBIs) aimed at reducing breast cancer screening barriers among the underserved, there is a lack of uptake or sub-optimal uptake of EBIs in community and clinical settings. This study evaluates a theoretically based, systematically designed implementation strategy to support adoption and implementation of a patient navigation-based intervention, called Peace of Mind Program (PMP), aimed at improving breast cancer screening among underserved women.

**Methods/design:**

The PMP will be offered to federally qualified health centers and charity clinics in the Greater Houston area using a non-randomized stepped wedge design. Due to practical constraints of implementing and adopting in the real-world, randomization of start times and blinding will not be used. Any potential confounding or bias will be controlled in the analysis. Outcomes such as appointment adherence, patient referral to diagnostics, time to diagnostic referral, patient referral to treatment, time to treatment referral, and budget impact of the intervention will be assessed. Assessment of constructs from the consolidated framework for implementation research (CFIR) will be assessed during implementation and at the end of the study (sustainment) from each participating clinic. Data will be analyzed using descriptive statistics (chi-square tests) and generalized estimating equations (GEE).

**Discussion:**

While parallel group randomized controlled trials (RCT) are considered the gold standard for evaluating EBI efficacy, withholding an effective EBI in practice can be both unethical and/or impractical. The stepped wedge design addresses this issue by enabling all clinics to eventually receive the EBI during the study and allowing each clinic to serve as its own control, while maintaining strong internal validity. We expect that the PMP will prove to be a feasible and successful strategy for reducing appointment no-shows in underserved women.

**Trial registration:**

Clinical trials registration number: NCT02296177

**Electronic supplementary material:**

The online version of this article (doi:10.1186/s13012-015-0334-x) contains supplementary material, which is available to authorized users.

## Background

### Importance of mammography adherence in underserved women

Breast cancer is the most common cancer and the second leading cause of cancer mortality among women in the USA [1], with roughly one in eight women developing breast cancer during their lifetime [2, 3]. Substantial breast cancer-related morbidity and mortality disparities persist among the underserved, particularly among African American women [1–6]. Disparities in breast cancer outcomes are due to lower mammography screening rates, lack of timely follow-up of abnormal results, and lack of timely treatment initiation among women with breast cancer. Stage at diagnosis is an important predictor of survival [7, 8]. Women who are diagnosed at a late stage are at higher risk for mortality from breast cancer, and are at risk for complications and overall poorer health outcomes [7, 8]. Underserved populations have consistently been shown to be at higher risk for late-stage diagnosis due to a combination of lower screening rates, missed appointments, and lack of timely referral to diagnostic evaluation and treatment [7–11]. Additionally, research has found that past mammography screening behavior is strongly correlated with current screening behavior, meaning that women who are off-schedule with screening are less likely to become adherent [12]. Appointment no-shows as they relate to the eventual completion of mammography screening have also been recently studied. Ontilo et al., 2013, found that women who missed a screening were more likely to be diagnosed at a later stage of cancer than women who attended [1]. In a 2012 study by Fayanju et al. of federally qualified health center (FQHC) patients in St. Louis, women who missed a screening appointment were significantly more likely to never receive definitive diagnosis or needed follow-up care [13]. A number of barriers prevent underserved women from obtaining timely screening, including patient-level socioeconomic differences like education, insurance, and income; differences in skills and qualifications of providers attending to different racial-ethnic groups; inadequacies in health care systems which provide care for the underserved; beliefs and misconceptions; and linguistic/communication barriers [8, 9, 14, 15]. A number of evidence-based intervention programs have been developed to address these barriers, yet disparities in breast cancer outcomes persist for underserved women. One reason for this may be the lack of uptake and use of evidence-based interventions in community and clinical settings that serve these women [16–20].

### The need for adoption and implementation support in clinics serving underserved populations

Dissemination and implementation processes for the uptake of evidence-based interventions (EBIs) remain poorly understood. The expertise of practitioners is often not used effectively, and implemented programs often do not ultimately reflect the needs of underserved communities within the specific context [17, 21, 22]. Particularly for interventions with minority communities, EBIs should be adopted and tailored at the community level by partnerships that include both researchers and practitioners, adding an additional level of complexity to the translation of these EBIs [23, 24]. Efforts to increase adoption and use of EBIs in community and clinical settings serving underserved women should target the safety net health care delivery system [25]. Within the safety net, primarily FQHCs and charity clinics provide comprehensive primary health care services for underserved communities regardless of ability to pay; this includes mammography screening [25, 26]. Additionally, the passage of the Affordable Care Act has significantly expanded the role of FQHCs who will be increasingly important in providing care for populations with a disproportionate burden of need [25]. Due to their client base, FQHCs are uniquely situated to reach and serve women in the most need for breast cancer screening [25]; however, FQHCs need assistance in planning and implementing EBIs [27, 28]. There is currently a lack of theoretically informed, well-documented, and evaluated implementation strategies for the translation of EBIs particularly within safety net clinical settings [4, 5]. Systematically designed implementation strategies are particularly important for the implementation of complex interventions—meaning interventions consisting of multiple behavioral, technological, and organizational components [3].

### Trial objectives

This study aims to address the research to practice gap and uses a theoretically based, strongly designed D&I strategy to support implementation of a breast cancer screening intervention aimed at promoting mammography adherence in underserved populations. Our patient navigation-based breast cancer screening intervention for the underserved is called the Peace of Mind Program (PMP). PMP is an EBI which was adapted from a National Cancer Institute Research tested intervention program and uses a tailored telephone counseling reminder call based on the Transtheoretical Model of Change to counsel patients through barriers to appointment attendance [20, 29–31]. PMP builds on our previous intervention protocol [30, 31] by adding intervention components to specifically support implementation within safety net clinics.

We hypothesize that the PMP intervention will improve mammography appointment adherence as compared to usual practice.

The specific objectives are:

1. To assess implementation of the intervention at the clinics.
2. To test the effectiveness of the PMP intervention in improving appointment adherence for screening mammograms.
3. To test the effectiveness of the PMP intervention in reducing the time it takes for a patient with abnormal results to receive follow-up and navigation to a diagnostic appointment and subsequent treatment initiation.
4. To assess the budget impact (cost savings) of the implementation of the PMP intervention from the perspective of the clinics.

## Methods

### Trial design

**Fig. 1:**
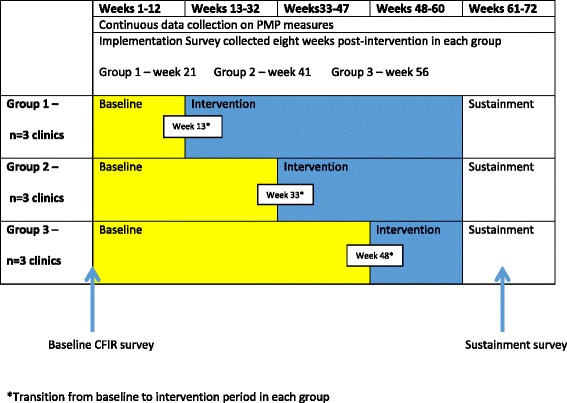
Stepped wedge trial design

We will conduct a non-randomized stepped wedge design as shown in Fig. 1. Each clinic will serve as its own control and will not have access to the PMP prior to implementation. Clinics will implement the PMP for up to 47 weeks depending on their group assignment (see Fig. 1). The trial will follow the CONSORT extension for stepped wedge randomized trials reporting guidelines (see Additional file 1). Insert reference: [Hemming K, Girling A, Haines T, et al. Protocol: Consort extension to stepped wedge cluster randomised controlled trial. 2014].

### Participants and settings

The PMP trial will be conducted in FQHCs and charity clinics who are members of the Breast Health Collaborative of Texas (BHCT) network. BHCT is a state-wide network of over 700 clinics and individuals with a shared mission to improve access to breast health services for all women in Texas.

### Practice inclusion criteria

In order to participate, clinics must (1) be members of BHCT within the Houston service area, (2) have a designation as a FQHC by HRSA or be a charity clinic which provides free or reduced cost care to underserved populations in their service area, (3) be serving women between the ages of 40–64 who are at or below 200 % of the federal poverty level for a family of four and who lack health insurance, and (4) engage in provision of mammography screening services at least six times per year and (5) women at the clinic must be in need of mammography screening and be scheduled for an upcoming appointment. Patients must have completed a clinical breast exam prior to their screening appointment.

### Practice exclusion criteria

There are no practice exclusion criteria if serving patients within the Houston service area.

### Intervention components

The PMP intervention program consists of the following components: a financial incentive of $7500 to the clinic for participation; funding for screening mammograms for women with documented financial need; coordination of increased screening within each clinic (the research team has established partnerships with local mobile mammography providers to assist in increasing screening capacity); 8 h of state certified training for staff in the EBI’s reminder phone call protocol [20, 30, 31]; stakeholder review of program adaptation and implementation materials; stakeholder meetings to troubleshoot implementation issues as they arise and to develop and disseminate e-newsletters and reports which highlight success stories and re-inforce desired behaviors; technical support for implementation (implementation manuals, clinic manuals, stakeholder manuals); and assistance in data tracking and reporting and budget impact analysis.

### Sample size

Sample size for the stepped wedge design is calculated using the formula of Woertman et al. 2013 [32], which corrects a standard sample size calculation for two independent proportions as follows:

$$ Nsw= Nu* DEsw $$

where *Nu* equals the uncorrected sample size estimate and *DEsw* is the design effect for the stepped wedge design.

$$ DEsw=1+\rho \left(ktn+bn-1\right)*\frac{3\left(1-p\right)}{\left(1+p\right)\left(\frac{1}{2ktn}+bn-1\right)2t\left(k-\frac{1}{k}\right)} $$

where *ρ* equals the intraclass correlation, *k* equals the number of steps, *b* equals the number of baseline measurements, *t* equals the number of intervention measurements, and *n* equals cluster size. The uncorrected sample size for a difference in two independent proportions assuming a baseline no-show rate of 35 % reduced to 28 %, an α of 0.05 and a power of 0.9 equals 924 per group. The average no-show rate and expected reduction in no-shows are based on pilot studies [20, 30]. The intraclass correlation coefficient (ICC), *p*, for mammography screening interventions has been shown to range from 0.0009 to 0.22 in community-based trials with an average ICC of 0.06 based on recent systematic reviews [33, 34]. Estimated sample sizes based on these parameters are summarized in Table 1. Based on the average reported ICC, the required sample size is 388 women. The required number of clusters is calculated as *Nsw/n* resulting in an effective sample size requirement of seven clusters (clinics) [32]. In order to account for potential attrition estimated based on the investigator’s pilot study, the sample size will be increased by 20 % to 466 women across nine clinics.

**Table 1:** Estimated sample sizes based on ICC reported values

*Nsw*	*Nu*	*DEsw*	*p* (ICC)
370	1848	0.20	0.006
388	1848	0.21	0.06
370	1848	0.20	0.1
333	1848	0.18	0.22

### Randomization and allocation

Clinic will be the unit of allocation in this study. Each clinic will be assigned a start date in groups of three (Fig. 1). Randomization of clinic start dates will not be done for practical reasons, including constraints in clinic operations (requirements for when they can or cannot start) and available screening dates (some clinics need additional time to reach the screening minimums required for study participation (*n* = 6)). The research team will assign a start date to each clinic based on these constraints.

### Blinding

Due to the nature of the intervention, it is not possible to blind the clinics or the research team in this study. However, outcome assessment is objective as we are using medical records to obtain screening outcomes. It is also not possible to blind the data analyst because the analysis is designed as a time series in which every clinic receives the intervention rather than a comparison between groups. The analysis will be pre-specified rather than blinded, and any analysis not pre-specified will be clearly delineated and considered hypothesis generating/exploratory rather than hypothesis testing/explanatory.

### Outcome measures

The primary outcome measure will be appointment adherence comparing the intervention period to the baseline period in each clinic. Appointment adherence will be considered using the following categories: no-show, not attended-called, late-screened, late-turned away, canceled/re-scheduled, and ineligible. A no-show will be considered a patient who makes no contact with the clinic prior to the appointment and does not attend on the day of mammography screening. A not attended-called is a patient who calls the clinic the day of the screening and asks to cancel or re-schedule (this may occur before or after their designated appointment time). Late-screened is a patient who arrives more than 30-min late for their appointment but is still seen by the mammography provided (e.g., worked in). Late-turned away is a patient who is more than 30-min late for their appointment and is turned away by the provider. A cancel or re-schedule is a patient who calls more than 24 h in advance to cancel or change their appointment date. An ineligible patient is a woman with symptoms who is ineligible to receive a screening mammogram and is turned away for a diagnostic evaluation or a woman who is turned away for incomplete paperwork. These categories are based on an assessment of the mammography providers’ and clinics’ behavior and the ways in which patient appointment outcomes are considered within a clinic system. Secondary measures will include patient referral to diagnostic (following screening) as a binary outcome and amount of time the referral took in days, patient referral to treatment as a binary outcome and the amount of time it took in days, and measures of implementation in each clinic based on the consolidated framework for implementation research (CFIR). A survey made up of scales assessing various CFIR constructs was developed by the Cancer Prevention and Control Research Network (CPCRN); we will use several scales from that survey in this study. These constructs will be measured at baseline (adoption), 8-week post-implementation, and following the end of the trial period (sustainment) [35–37]. Because randomization of start dates is not possible, we will control for potential confounders in the analysis of the data. Control variables may include time to intervention start date in each clinic, clinic size, staffing, and whether or not the clinic had a pre-existing patient navigation program for breast cancer screening, patient demographics, region served, and infrastructure characteristics (e.g., funding) (taken from the Health Resources and Services Administration Uniform Data System (UDS) reports for FQHCs and our economic survey).

### Data collection

Data collection protocols will be standardized across all clinics. In clinics with the capability to collect and report data from their electronic health record (EHR), the research team will seek to adapt the existing system for data collection. In clinics where the EHR is not easily modified or one is not available, data collection will be supported via the use of a customized Redcap database. The database will have a front-facing data entry interface which is user friendly and supports accurate data collection for statistical analysis. The patient navigator (or implementer) in each clinic will be responsible for tracking upcoming patient mammography appointments, reminder phone call attempts, patient consent for the trial, and the EBI screening questions and barriers counseled during the phone call. Appointment attendance, referral to diagnostic mammography, and treatment data will be abstracted from medical records in each clinic. For the budget impact analysis, two surveys will be used to assess the FQHC/clinic resource utilization before and after the implementation of the intervention. We are also collecting patient resource utilization data to understand what parts of patient costs are being subsidized by the FQHCs/clinics and to also ensure availability of cost information from patient perspective in case an economic evaluation from societal perspective is deemed important.

### Statistical methods

Descriptive statistics will be calculated for all primary and secondary measures. Following descriptive analysis, regression modeling will be used to evaluate outcomes while allowing for control of potential confounders (e.g., time, clinic size, staffing, and whether or not the clinic had a pre-existing patient navigation program for breast cancer screening). Due to the correlated nature of the data and repeated observations taken on each clinic, generalized estimating equations (GEE) will be used to model outcomes while allowing us to control for potential confounders [38, 39]. GEE is used to estimate the parameters from a generalized linear model when there is possible correlation between outcomes [38, 39]. GEE is a population averaged model that estimates the average response from a change in a covariate over the population rather than individual [38, 39]. GEE model estimates are less sensitive than generalized linear mixed models to the specification of the variance structure in the data making them a flexible choice [38, 39]. Data on appointment adherence will be analyzed using various sub-groups to evaluate the intervention effect. For example, no-shows and not attended-called may be combined to consider a more stringent definition of a no-show. They will also be analyzed separately. We expect the intervention to have an effect on the no-shows, not attended-called, late-screened, late-turned away and ineligible categories and will evaluate that effect separately. Sub-categories will be compared using chi-square tests to determine differences in outcomes between groups (control and intervention). Data from the CFIR survey will be analyzed using descriptive statistics and in the regression models to evaluate which constructs are related to implementation outcomes. Data analysis will be conducted in Stata (Stata Corp., College Station, TX).

### Budget impact analysis

The annual budget impact analysis (BIA) will be performed from the perspective of the clinics [40, 41]. The aim of the BIA is to assess the additional cost each clinic will incur to implement and sustain the EBI. The population of interest for the BIA will be the patients served by each clinic who are eligible to receive a mammogram for each year under study. Each patient will be the unit of analysis for the purpose of estimating the volume and resources required. The dollar value and the price rates for the expenditures incurred by each clinic will be standardized at the final year of the study for the BIA. This standardization will circumvent the need to use discount factors and inflation adjustment for the annual BIA values pertaining to each year of the study. The average cost per year will be computed for three different time periods:

1. The pre-intervention/control period, which will give us a distribution of cost for the screening reminder or navigation systems currently used by the clinics. We expect to find a wide variety of patient navigation or screening reminder systems during this period. Many clinics might have no mechanism in place for navigation or screening reminders for mammogram eligible patients.
2. The intervention period, which will help estimate the initial investment and implementation cost associated with the EBI. Cost associated with this period will be the highest among the three periods and will provide an estimate of the upfront expenses for any new clinic establishing an EBI program. It will consists of the fixed cost of training the navigators on the EBI methods, cost associated with structural changes to the clinic’s EMR, which will help link the patient records with the appropriate EBI scripts, and cost of developing web-based scripts for patient navigation. Based on the volume of patients served, the intervention period cost will also include the variable cost of hiring additional navigators for implementing the EBI. The navigators will not only help improve the provision of mammograms but also facilitate diagnostic test administration and treatment initiation for patients who need these. The patient volume and amount of time required per patient will be obtained during the implementation period from each of the clinics. The initial time required to navigate a patient and improve adherence to the mammogram screen will be similar between patients. However, if the patient has a positive mammogram, the patient will require further navigation to obtain a diagnostic test (biopsy). Patients with positive diagnostic tests will also require navigation for treatment initiation. Although the study’s primary goal is to improve screening adherence and reduce screening “no-show” rates, the navigators are also involved with diagnostic and treatment navigation when required by the patient. Hence, cost of those tasks will also be included in the analysis.
3. The post-intervention period, which will help estimate the steady state cost for each clinic with no external support for the EBI. Post-intervention provision of EBI and associated costs will be obtained 3 months after the end of the study.

### Trial status

The trial has completed enrollment of the first wave of clinics (*n* = 9) and is currently collecting baseline measures at these locations. Baseline measures include appointment adherence outcomes as described above, the clinic and patient resource utilization survey for the budget impact analysis, and CFIR survey. All participating clinics have completed the baseline CFIR survey. Based on the number of mammography screening drives at each clinic, we anticipate enrolling approximately 2200 women into the first wave of the trial.

## Discussion

### Rationale for the stepped wedge design

While parallel group randomized controlled trials (RCT) are considered the gold standard for evaluating EBI efficacy, withholding an effective EBI in practice can be seen as unethical and/or impractical [42–45] . The stepped wedge design addresses this issue by enabling all clinics to eventually receive the EBI during the study and allowing each clinic to serve as its own control, while maintaining strong internal validity [42–45]. Stepped wedge designs are particularly useful when the intervention is thought to do more good than harm and when an RCT is impractical, as is the case in this study (e.g., financial, logistical reasons) [45–47].

### Anticipated challenges

Despite the advantages to using a stepped wedge design for the PMP trial, we anticipate a number of challenges. The staggered entry into the intervention period means that clinics assigned to a later start date will start the intervention 48 weeks after their initial agreement to adopt the program. This may mean that the motivations for initially agreeing to adopt the program may no longer apply. We also anticipate that clinics may be frustrated by having to wait to start the program even if they stay motivated to participate. With that in mind, we defined a strategy to maintain clinic interest and engagement while they are waiting to start PMP. Each clinic will participate in a stakeholder committee where they will review implementation plans, handbooks, and undergo intervention training. The schedule for the stakeholder committee is staggered such that the later groups spend more time working in this venue in order to keep them engaged. A further potential problem in stepped wedge designs is contamination between clinics and those waiting for the intervention. It is possible that some clinics may change their procedures based on what they hear or see with other participating clinics. However, we expect such effects to be mild because meaningful and sustained improvements in mammography appointment adherence require systematic and prolonged effort that is unlikely to occur before the PMP training is completed.

### Ethical approval

This study has received approval from the Institutional Review Board at the University of Texas Health Science Center Houston. The protocol number is HSC-SPH-14-0269. Verbal consent will be obtained for patients participating in the EBI reminder phone calls. Written consent will be obtained for economic data collection from patients.

## Additional file

Additional file 1: Table S1. CONSORT 2010 checklist of information to include when reporting a cluster randomized trial. (DOCX 21.9 KB)
